# A Levee to the Flood: Pre-injury Neuroinflammation and Immune Stress Influence Traumatic Brain Injury Outcome

**DOI:** 10.3389/fnagi.2021.788055

**Published:** 2022-01-12

**Authors:** Samuel Houle, Olga N. Kokiko-Cochran

**Affiliations:** ^1^Department of Neuroscience, College of Medicine, The Ohio State University, Columbus, OH, United States; ^2^Institute for Behavioral Medicine Research, Neurological Institute, The Ohio State University, Columbus, OH, United States

**Keywords:** traumatic brain injury, inflammation, microglia, mitochondria, Alzheimer's disease

## Abstract

Increasing evidence demonstrates that aging influences the brain's response to traumatic brain injury (TBI), setting the stage for neurodegenerative pathology like Alzheimer's disease (AD). This topic is often dominated by discussions of post-injury aging and inflammation, which can diminish the consideration of those same factors before TBI. In fact, pre-TBI aging and inflammation may be just as critical in mediating outcomes. For example, elderly individuals suffer from the highest rates of TBI of all severities. Additionally, pre-injury immune challenges or stressors may alter pathology and outcome independent of age. The inflammatory response to TBI is malleable and influenced by previous, coincident, and subsequent immune insults. Therefore, pre-existing conditions that elicit or include an inflammatory response could substantially influence the brain's ability to respond to traumatic injury and ultimately affect chronic outcome. The purpose of this review is to detail how age-related cellular and molecular changes, as well as genetic risk variants for AD affect the neuroinflammatory response to TBI. First, we will review the sources and pathology of neuroinflammation following TBI. Then, we will highlight the significance of age-related, endogenous sources of inflammation, including changes in cytokine expression, reactive oxygen species processing, and mitochondrial function. Heightened focus is placed on the mitochondria as an integral link between inflammation and various genetic risk factors for AD. Together, this review will compile current clinical and experimental research to highlight how pre-existing inflammatory changes associated with infection and stress, aging, and genetic risk factors can alter response to TBI.

## Introduction

Traumatic brain injury (TBI) is a growing healthcare burden worldwide. In the United State alone, more than 1.5 million people sustain a TBI each year, resulting in more than 200,000 hospitalizations (Goyal and Yadav, 2020). These injuries only account for what is recorded from individuals who seek medical attention. Analysis correcting for underreporting estimates the true number of TBIs per year to be ~3.8 million, leading many to refer to TBI as a “silent epidemic” (Laskowski et al., 2015). Injuries often include contusions and diffuse axonal injury, both of which stem from mechanical trauma to the head. Commonly, this mechanical trauma occurs as a result of impact injuries, including falls, assault, or rotational/vestibular injuries associated with motor vehicle accidents. Advances in healthcare have improved survival rates after TBI however, estimates suggest that roughly 5.3 million people experience chronic, TBI-related disabilities (Faul and Coronado, 2015). Among the most common disabilities are seizures, cognitive impairment, emotional dysregulation, and sleep disruptions (Ahmed et al., 2017). Notably, these estimates do not capture the financial strain from medical costs and lost wages or increased stress on caregivers (Taylor et al., 2017), which further contributes to financial burden. When considering the frequency of TBI, combined with physical, economic, and emotional toll that these injuries can inflict, greater investigation and understanding of the pathology is needed.

TBI results in two phases of injury, primary and secondary, each having their own distinct pathogenesis. Primary injury occurs directly as a result of mechanical trauma to the head or neck. This acute phase of injury is characterized by the immediate death of nervous tissue, which is irreversible. Tissue damage in the primary acute phase of injury elicits an immune response driven upon activation of microglia, the resident immune cells of the central nervous system (CNS), by cytokines and chemokines (Shi et al., 2019). Inflammation drives secondary injury following TBI, which can persist chronically and is not limited to the focal point of injury. In secondary injury, inflammation spreads to distal brain regions that are otherwise unaffected by primary injury. While neuroinflammation is thought to be neuroprotective during primary injury, chronic activation may be a key contributor to pathology during secondary injury. Immune challenges including TBI induce microglial “priming,” a state in which microglia are hyperresponsive to subsequent immune challenge. While in the primed state, microglia have a lower threshold for activation and are more susceptible to hyperactivation (Norden and Godbout, 2013; Witcher et al., 2015). Microglia are primed following TBI, and post-injury, secondary immune stressors complicate functional recovery (Norden et al., 2015). Despite clinical significance, the effects of pre-TBI inflammation on injury outcome is less studied and understood. Sources of pre-TBI inflammatory load are heterogenous and have different patterns of immune activation throughout the body. In these scenarios, TBI, in effect, becomes the secondary immune challenge to a brain that is already experiencing either acute or chronic inflammation.

This review aims to describe the neuroinflammatory profiles of several potential sources of such inflammation. For example, age-associated changes in cytokine expression, reactive oxygen species (ROS) processing, and mitochondrial function can antagonize the CNS environment prior to injury, damaging the brain's ability to respond to and recover from TBI. Emphasis will be placed on the intermediary role of mitochondria in CNS inflammation and how changes to mitochondrial function could impair the immune response to TBI. Furthermore, we will review how several genetic variants linked to Alzheimer's disease can influence these same pathways and could alter the neuroinflammatory landscape prior to TBI. Coincidentally, microglial mitochondria are uniquely affected by many of these variants. Therefore, the bulk of this review will focus on endogenous sources of altered inflammation. We will also briefly highlight current literature investigating exogenous pre-injury stressors, namely infection and environmental stress. Together, this review will compile current research to highlight how pre-existing inflammatory changes can alter response to TBI.

## Etiology of TBI

TBI occurs as a result of trauma to the head or neck. This injury is considered heterogeneous, having many classifications, causes, and severity levels. TBI can be classified as focal or diffuse. Focal injuries occur when a foreign object or piece of skull penetrates the brain, or from increased pressure generated from hemorrhages and acute ischemic events (Weber and Maas, 2007). Damage caused by focal injuries is commonly limited to the site of injury with tissue death and neuroinflammation in the cortex ipsilateral to injury (Allahyari and Garcia, 2015; Jin et al., 2016). Experimental models for this type of injury primarily consist of stab-wound and controlled-cortical impact (CCI), although ballistic-like models have been developed more recently (Cernak et al., 2014).

Diffuse injuries occur when the head or neck experience a sudden mechanical trauma causing the brain to impact the interior of the skull. While most immediate tissue death is centralized to the injury site, global changes in inflammation and HPA axis function have been observed using models for diffuse injury and can persist chronically (Loane et al., 2014; Tapp et al., 2019). Causes of diffuse TBI are largely dependent on age at the time of injury. In adolescents, common sources of diffuse TBI are car accidents, falls, and sports injuries while TBIs in aged populations originate mostly from falls. Injuries from violence also constitute a significant population, between 7 and 10% of closed head injuries are attributable to random assaults, intimate partner violence and domestic violence, and blast injuries (Maas et al., 2008).

The severity of TBI is further described as mild, moderate, and severe. Mild TBI (mTBI) is the most common form of brain injury while moderate TBI accounts for 11% of TBIs (Dewan et al., 2019). While less common than mTBI, moderate TBI presents an interesting opportunity for study. Moderate TBI impacts a significant 7.5 million people globally and the acute and chronic effects are more pronounced than the effects of mild injuries, with global CNS effects lasting for decades following injury (Ramlackhansingh et al., 2011; Johnson et al., 2013; Dewan et al., 2019). With a lower death rate than severe TBI, moderate TBI also creates a unique population of survivors who continue to live for decades after injury while having to manage chronic symptoms. Thus, understanding factors that can impact the severity and longevity of post-TBI complications is extremely consequential in a healthcare setting. Therefore, this review will primarily focus on studies describing single incident, moderate TBI.

## Pathogenesis of Primary and Secondary Injury

Primary injury is categorized by immediate axon damage and cell death following TBI that is not preventable or treatable. This phase of injury is closely associated with the acute symptoms of TBI regardless of severity. Damage occurs to neurons at both a cellular and macroscopic level. At the cellular level following moderate TBI, neurons can have damaged membranes and dysfunction of ion channels. These two factors can lead to influxes of Ca^2+^ ions and extracellular proteins that drive necrosis near the injury site. This cell death is irreversible and results in lasting cognitive impairments. At the macroscopic level, axons are injured by the sheering forces of rapid acceleration and by the death of myelinating oligodendrocytes (Bramlett et al., 1997; Flygt et al., 2016). Shearing of axons can impair neuronal transmission disrupting global neural networks (Wolf and Koch, 2016).

Following moderate TBI, a series of biochemical changes occur in the brain including: the production of free radicals and lipid peroxidation, mitochondrial dysfunction, cell necrosis, and blood brain barrier (BBB) break down (Alam et al., 2020). These biochemical changes, as well as the release of damage-associated molecular patterns (DAMPs) from damaged cells and mitochondria, stimulate the production of cytokines in nearby cells. Cellular and mitochondrial DAMPs including free DNA and mitochondrial DNA (mtDNA), cytokines, ATP, potassium, and larger molecules like S100B can activate multiple pattern recognition receptors (PRR) including; toll-like receptors (TLR), inflammasomes, and receptor for advanced glycation end-products (RAGE) on cells and microglia (Vos et al., 2010; Zhang et al., 2010; Goulopoulou et al., 2012; Balança et al., 2021). Additionally, at the site of injury, immune cells recruited from the periphery including monocytes, neutrophils, and lymphocytes contribute to the release of DAMPs (Wang and Shuaib, 2002).

Secondary injury is characterized by continuing cellular death *via* apoptosis. This phase succeeds primary injury and is not attributable to the mechanical forces of head trauma but instead, to changes in the biochemical makeup of the brain and neuroinflammation. In moderate TBI this stage of injury is marked by increased inflammation compared to mTBI that contributes to chronic symptoms and complications. Activation of PRRs stimulates the production of pro-inflammatory cytokines *via* the myeloid differentiation primary response 88 (MyD88)/extracellular signal-regulated kinase (ERK)/nuclear factor kappa B (NF-κB) signaling pathway (Fitzgerald et al., 2001; Diomede et al., 2017). Upon sensing release of these factors, microglial reactivity is enhanced. As a result, microglia take on reactive profiles by exhibiting increased phagocytic activity, and increased production and release of pro-inflammatory molecules. In addition, following TBI, microglial morphology can change to facilitate movement through the parenchyma. De-ramified “bushy” morphologies have been observed in animal models of diffuse and focal TBI while more swollen “ameboid” characteristics can appear in focal injuries where tissue damage is more extensive (Cao et al., 2012; Donat et al., 2017; Caplan et al., 2020). In the context of neurodegenerative diseases and brain injuries, this reactive state can persist chronically with negative consequences including neurodegeneration and cognitive/behavioral deficits (Hauss-Wegrzyniak et al., 1998; Schlachetzki and Hüll, 2009; Ramlackhansingh et al., 2011; Norden and Godbout, 2013; Loane et al., 2014). The intent of this neuroinflammatory response is neuroprotective, initially recruiting neutrophils to clear cellular debris from the brain. This effect is short lived with neutrophil populations peaking 48 hours post injury (Carlos et al., 1997). However, microglia that are activated by the damage signals continue to stimulate neuroinflammation both at the site of injury and throughout the rest of the brain for days to years after TBI (Simon et al., 2017).

## Microglial Response to TBI

Microglial progenitor cells derive from myeloid tissue and populate the emerging CNS during embryonic and fetal development (Chan et al., 2007). Mature microglia have long life-spans with low turnover. Under typical conditions, microglia provide surveillance of the CNS environment by extending processes through the parenchyma on order to search for danger and chemotaxic signals (Nimmerjahn et al., 2005). When signals are sensed by surveying cells, microglia become both genotypically and phenotypically responsive. Phenotypic activation is defined by a de-ramified, ameboid shape, in which processes are retracted. This allows for easier taxis through the CNS parenchyma. Genotypic activation has proven more complex. A recent study using 96 different stimulators of microglial activation found 33 distinct activation expression modules. Of these 33 expression patterns, 15 could be stimulated by lipopolysaccharide (LPS) a component of gram-negative bacterial membranes and common model for bacterial infection (Cho et al., 2019). This indicates that there is a spectrum of microglial reactivity and different stimulants of immune activation induce unique profiles of activation. When chronically exposed to pathogens, damaged tissue, or stress, microglia can become primed meaning that they have lower thresholds for activation and demonstrate exaggerated responses to stimuli.

To investigate the role of microglial reactivity in injury pathology several studies have depleted microglia from the brain prior to, or after TBI. Myeloid-lineage cells including macrophages and microglia depend on colony stimulating factor signaling for cell viability and proliferation. Colony stimulating factor-1 receptor (CSF1R) is an important part of this signaling pathway. Inhibition of the receptor by dietary CSF1R antagonists results in the depletion of up to 99% of all CNS microglia, while animals are on the drug. Microglial repopulation will take place within a week following cessation of drug administration. These microglia do not repopulate from infiltrating monocytes, instead it has been suggested that microglia arise from a progenitor or progenitor-like cell population in the brain (Elmore et al., 2014). Recent analysis suggests new microglia emerge from an existing population of MAC2^+^, microglia progenitor-like cells that are resistant to CSF1R inhibition (Zhan et al., 2020). Importantly, these new cells are indistinguishable from the microglia that were present prior to depopulation, allowing for a detailed look into the role that these cells play in TBI pathology (Elmore et al., 2014; Rice et al., 2017). Depletion of microglia in mice *via* inhibition of CSF1R 1 month following moderate controlled cortical impact (CCI) displayed several effects. These included decreased lesion volume, increased neuron density in the cortex and dentate gyrus, and increased the proportion of ramified microglia. Delayed microglial depletion also decreased NOX2 and CD68 expression in microglia, indicating decreased microglial activity which correlated with functional improvements in Y-maze and MWM performance (Henry et al., 2020). More recently, a study by Witcher et al., examined how eliminating microglia prior to a moderate midline-fluid percussion injury (mFPI) changed gene expression in the cortex. This study found that increases in neuroinflammation-related proteins CD24, CD68, TLR4 and triggering receptor expressed on myeloid cells (TREM) 2, type-1 interferons, and chemokine-related transcripts observed in injured mice 7 days post injury (dpi) were attenuated by microglia depletion. Additionally, the authors found that neuronal pathology was alleviated by depletion as well. TBI-related transcriptional changes in neurons were reversed when microglia were cleared. These genetic changes correlated with the recovery of TBI-related deficits in novel object recognition (NOR) task for spatial memory (Witcher et al., 2021). Together, these findings indicate that in an inflammatory state microglia have the ability to directly contribute to neuropathology and impact neurological recovery and outlook after TBI. Thus, microglial reactivity is directly implicated as a key player in secondary injury pathology.

## Microglial Priming and Responses to Secondary Immune Challenges

Primed microglia continually express pro-inflammatory transcriptomes and morphologies and are hyper-responsive to secondary immune challenges (Streit et al., 2004; Holtman et al., 2015). When primed, microglia have a lower threshold for reactivity and display exaggerated responses once activated. This phenomenon has been confirmed in aging mouse models (18–20 months old) injected with LPS that found exaggerated production of pro-inflammatory cytokines IL-1β and IL-10, and major histocompatibility complex (MHC) II upregulation in microglia (Henry et al., 2009). This indicates that primed microglia adopt a hyperactive phenotype when confronted with secondary immune stressors. Just like aging, TBI can lead to primed microglia that exist at chronic timepoints. Histochemical analysis of brain tissue from clinical TBI patients showed an increase in moderate pathology at subacute (up to 1 year), and long term (over 1 year) timepoints. This pathology included chronically reactive microglia and decreased white matter integrity (Johnson et al., 2013).

Subsequent immune challenges may synergize with brain injury-induced neuroinflammation to exaggerate these deficits. For example, several studies show that simulated immune challenges following TBI such as infection, stress (sleep disruption), exacerbate neuroinflammation, and functional impairment (Hang et al., 2004; Tapp et al., 2020). Additionally, existing factors at time of injury such as immunosenescence, and concurrent polytrauma can impair outcomes to TBI (Sun et al., 2018; Suto et al., 2018). Regardless of the source, multiple studies have shown that secondary immune challenges can interact with TBI to worsen inflammation and behavioral recovery. Injections of LPS following moderate TBI in both mice and rat models led to exaggerated cytokine production compared to non-treated TBI animals and induced sickness-like behaviors including anxiety, social withdraw and anhedonia (Hang et al., 2004; Fenn et al., 2014). Similarly, stressing mice by foot shock after dual closed-head injuries led to increased depressive-like behaviors (Klemenhagen et al., 2013). Lastly, even psychological stressors display this interaction effect. Disrupting mouse sleep following moderate lateral fluid percussion injury exacerbates the inflammatory response, resulting in persistent glial fibrillary acidic protein (GFAP) expression, and TREM2, and TLR4 activation at 7 dpi (Tapp et al., 2020).

**Figure 1:**
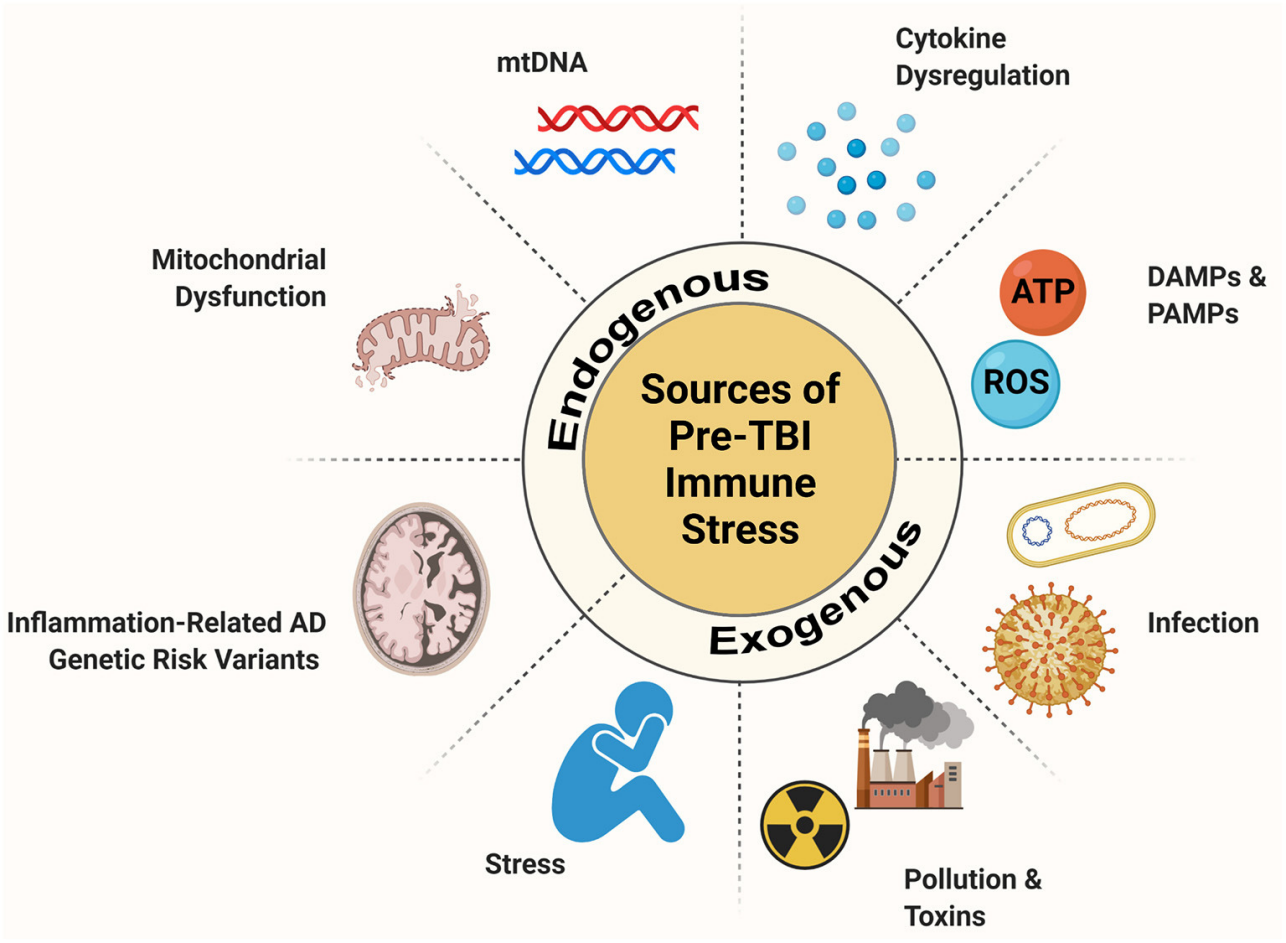
Source of pre-injury immune stress. When the CNS experiences an immune stressor, a robust inflammatory response takes place. Microglia, the resident immune cells of the brain, are key players in this process. When microglia respond to an immune challenge they enter a primed state where they are more readily reactive and display exaggerated responses. If microglia are primmed by a pre-TBI inflammatory challenge this has the potential to alter the inflammatory response to injury. Pre-TBI immune challenges can stem from exogenous sources like infection, environmental stressors like pollution and stress, and psychological or social stress. These, as well as endogenous sources such pre-injury cytokine dysregulation, mitochondrial dysfunction, and release of danger signals and genetic changes that impact inflammatory regulation can impair aspects of the CNS immune system. These factors can pre-set the immune system to a differential reaction to TBI with possible consequences for chronic outlook and recovery.

The stacking of immune challenges appears to contribute to worse neuropsychiatric outcomes following TBI, leading researchers to hypothesize that hyperactive microglia constitute a link between TBI and future neurodegenerative pathology (Loane and Kumar, 2016; Kokiko-Cochran and Godbout, 2018). While much emphasis has been placed on studying the effects of concomitant and post-TBI secondary immune stressors, the importance of pre-TBI immune activation has been underappreciated. Pre-TBI immune stress and neuroinflammation and have multiple sources (Figure 1). In fact, while most evidence concludes that increasing immune load following injury has a negative impact on recovery, the evidence on how pre-injury factors interact is incongruent. Therefore, it is important for the field to take stock of our current understanding of how various source of inflammatory load can predispose the brain to alternate outcome following TBI.

## Prior Infection

Pre-injury inflammation in certain contexts has demonstrated neuroprotective abilities. It is possible for a prior immune challenge to actually improve the CNS's response to a second immune challenge. This neuroprotective preconditioning has been described both within and outside of TBI literature. LPS induces an immune response *via* stimulation of TLR4 receptors when injected, modeling the immune response to a bacterial infection. In ischemia literature, LPS doses (0.5–0.9 mg/kg) delivered prior to an ischemic event protected the CNS, decreasing the size of a subsequent infarct and functionally leading to lower death rates (Tasaki et al., 1997; Rosenzweig et al., 2007). In the context of TBI, several models of prior infection have demonstrated preconditioning. In a rat, weight-drop model of TBI, preconditioning with LPS (0.2 mg/kg) 7 days prior to injury attenuated glial activation and neuron loss (Turner et al., 2017). Similar findings in a rat CCI model found that pretreatment with LPS (0.1/0.5 mg/kg) 5 days prior to injury, significantly decreased neuronal apoptosis in the hippocampus and decreased overall caspase activity (Eslami et al., 2020).

Other models of infection provide similar results. Polyinosinic-polycytidylic acid (poly I:C) is a synthetic structure analogous to mismatched double stranded RNA. This structure closely resembles viral RNA, making it a common model for viral infection. Like LPS, poly I:C is recognized by TLR's, specifically TLR3 (Kadowaki et al., 2001). In a rat model of ischemic injury, a single dose (0.3 mg/kg) of poly I:C delivered prior to middle cerebral artery occlusion (MCAO) was sufficient to precondition the CNS leading to a decrease in infarct size and volume and improvements in post MCAO neurological scores. Notably post-MCAO levels of TNF-a and IL-6 were also lower in poly I:C treated mice (Pan et al., 2012). These findings mirror those observed using LPS to precondition the CNS prior to ischemia. However, there is currently a lack of literature investigating whether pre-TBI poly I:C would provide similar neuroprotective preconditioning to the CNS.

Further investigation into this area would help provide clarification on whether the neuroprotective aspect of preconditioning is specific to particular TLRs and their downstream effects. For example, the role of TLR's in stimulating changes in cytokine regulation is particularly interesting. Stimulation of TLR2, TLR4, TLR7, and TLR8 all induce changes in cytokine expression, namely increases in pro-inflammatory IL-1β and TNF-α, and decreases in anti-inflammatory IL-6 expression (Lavieri et al., 2014). Current literature on the mechanisms of neuroprotective preconditioning indicates roles for TLR4, and the downstream MyD88 and TIR-domain-containing adapter-inducing interferon-β (TRIF) pathways. Specifically, Vartanian et al., found that mice that were preconditioned with LPS (0.8 mg/kg) had an increase in mRNA for NF-κB inhibitors *Ship1, Tollip*, and *p105*, corresponding with a decrease in NF-κB activity 24 h post injury. In contrast, Vartanian et al., found an increase in interferon regulatory factor 3 (IRF3) indicating a shift from MyD88/NF-κB to TRIF/IRF3 signaling after LPS exposure. Additionally, MyD88^−/−^ but not TRIF^−/−^ mice had decreased infarct sizes after MCAO retreated with LPS (Vartanian et al., 2011). These data present a mechanism where a shift toward TRIF/IRF3 signaling confers neuroprotection from future insult, possibly by attenuating NF-κB mediated pro-inflammatory cytokine production. This allows for greater balance between pro and anti-inflammatory NF-κB signaling post-LPS exposure (Vartanian et al., 2011; Sangaran et al., 2021). Despite neuroprotective preconditioning being heavily tied to TLR activation and subsequent cytokine production, the role of TLRs in pre-TBI inflammation may not be definite. Later in this review we will present data that shows knocking out TLRs and knocking down pro-inflammatory cytokine activity actually attenuates secondary injury (Bermpohl et al., 2007; Ahmad et al., 2013; Chung et al., 2019).

## Age-Related Inflammation

Aging itself is associated with higher levels of transient inflammation (Godbout and Johnson, 2009). While the source of inflammation in aging are varied and will be reviewed in their own right, the effect of overall pre-TBI aging has been investigated with differing results. In a rat model of moderate mFPI, Rowe et al. (2016) found that rats injured in adolescence (PND 17-35) displayed more motor and cognitive deficits but less anxiety-like behavior than rats injured in adulthood (2, 4, or 6 months). Recent studies have demonstrated more profound effects of age on TBI outcomes. In a rat model for moderate lateral FPI, Sun M. et al. (2019) found that aged rats (1 year) had decreased beam walk performance, longer search times in Morris water maze (MWM), and suppressed myeloid and microglial infiltration following injury compared to young injured animals. Age at time of injury also impacts neuropathological hallmarks in the brain and alters long term gliosis. Doust et al., found that aged rats (10 months), that had received a recent (2, 4, or 6 months) moderate mFPI had increased dendrite neurofilament pathology and higher CD68 activity in microglia. Additionally rats that received the brain injury earlier in life (PND 17–35) had higher colocalization between TREM2 and microglia in the hippocampus (Doust et al., 2021). These indicate that age at time of injury impacts neuropathology but even brain injuries received earlier in life can continue to interact with aging neuroinflammation. These studies as well as clinical evidence suggests that adults benefit from completed neurodevelopment in their response to TBI while elderly populations are particularly susceptible to TBI. Longitudinal studies found that both the existence of mild cognitive impairment and progressive cognitive decline after TBI were influenced by age at time of injury (Himanen et al., 2006; Ziebell et al., 2017). These finding have been corroborated in animal models. CCI in 24 month aged mice vs. 3 months old mice produced exaggerated microglial responses and led to increased lesions size while 18 months old mice had decreased performance on motor tasks compared to control groups (Kumar et al., 2013; Ritzel et al., 2019).

Together this evidence suggests that the severity and nature of TBI sequalae are at least in part influenced by age at time of injury. Several factors of aging likely play a role in this including immunosenescence, oxidative stress, and mitochondrial dysfunction. While these factors are prevalent during the aging process, they can also occur due to non-aging related inflammation. Independent of age, certain genetic variants associated with AD development and even environmental stressors can alter neuroinflammation prior to TBI. For both sources of pre-TBI inflammation mitochondria play a key role in modulating appropriate microglial responses. Therefore, this review will investigate how both aging and non-aging factors can alter the immune profile of the brain prior to injury and how this may impact the pathogenesis of secondary injury with a focus on the role of mitochondria in these processes.

### Cytokines

Cytokines are involved in a myriad of roles following an immune challenge in the CNS. While generally classified as pro- or anti-inflammatory, this dichotomy hides the more complex relationship that cytokines have with both neurons and glia. In addition to modulating microglial reactivity, many cytokines can also recruit immune cells into the brain and alter BBB permeability. Importantly, cytokines also regulate the production of other cytokines in both homeostatic and inflammatory directions (Dugue et al., 2017). Thus, cytokines play an important role in the general state and adaptability of the CNS. Cytokine dysregulation contributes to the rise in transient neuroinflammation with age. Both humans (86–94 years) and rodents (14 months) display increased levels of pro-inflammatory cytokines like IL-1β, IL-6, and type-1 interferons (IFN) but not similar increases in anti-inflammatory cytokines, like IL-10 compared to younger humans (32–59 years) and rats (2.5 months) (Forsey et al., 2003; Xin et al., 2011). These higher levels are likely driven by a variety of factors. Factors that will be discussed later including ROS production and oxidative stress increase with age and trigger the MyD88/ERK/NF-κB signaling pathway to induce transcription of pro-inflammatory cytokines (Rea et al., 2018). In addition, evidence indicated that senescent cells accumulate with age and can adopt a phenotype where they perpetually produce cytokines *via* NF-κB activation (Salminen et al., 2012). At the same time, multiple studies have shown that both TBI outcomes are worse in aged TBI models (1 year old rats), that cytokine levels are elevated after injury in both humans and rodents, and that these elevated cytokine levels are indicative of outcome and cognitive performance post-injury (Chiu et al., 2016; Sun M. et al., 2019; Sun Y. et al., 2019). Protein levels are elevated for IL-1β, IL-12, and IFN-γ for several days post injury regardless of injury severity, indicating they may contribute to the progression of secondary injury (Lagraoui et al., 2012).

Currently, there is a lack of direct evidence of how preinjury cytokine levels, in isolation from other aging factors, may impact outcome. However, several studies have prophylactically targeted pro- and anti-inflammatory cytokine levels to attenuate aspects of TBI pathogenesis. Animal knockout models for proinflammatory cytokines generally mitigate aspects of TBI pathology. By knocking out IL-1 receptor type 1 (IL-1R1) in adult mice Chung et al. (2019) were able to reverse spatial memory deficits observed in MWM and Y maze following closed-head impact model of mild TBI. Similarly, *Tnf*−α/*Fas*^−/−^ displayed improved performance on MWM following TBI in a mouse CCI model (Bermpohl et al., 2007). IFN-β levels are implicated in both acute and chronic TBI pathology. Inhibition of type-1 interferon signaling through constitutive IFN alpha receptor 1 (IFNAR1) knockout or *via* knockout of stimulator of interferon genes (STING) alleviated acute TBI pathology, attenuating expression of TNF-α, IL-1β, and IL-6, and reduced lesion volume in a mouse CCI model 24 h post-injury (Karve et al., 2016; Abdullah et al., 2018). IFN-β deficiency also provides protection from secondary injury. In a mouse model of moderate CCI, *Ifn*−β^−/−^ mice had suppressed expression of TNF-α, IL-1β, IL-6, CCL5, and NADPH oxidase 2 (NOX2). IFN-β deficiency also improved long term neurological recovery, improving performance on Y-maze (8 dpi), NOR (17–18 dpi), and beam-walk (0–28 dpi) (Barrett et al., 2020). These palliative effects are not universal. Ley et al. (2011) found that IL-6 KO mice subjected to TBI *via* CCI had elevated IL-1β levels performed worse in open field and rotarod behavioral tasks. This underscores the important role that cytokines play in regulating cytokine production. Additionally, IL-6 in particular is important as it promotes reparative functions of microglia post-TBI. In a mouse CCI model, Willis et al. (2020) found that depleting and repopulating microglia during the acute phase of injury helped promote neurogenesis in the hippocampus and this was dependent on IL-6/IL-6Ra signaling. This demonstrates that proinflammatory cytokines may also have important repair signaling functions after TBI. Taken together, these studies demonstrate the cytokines have an outsized role in aging and TBI-related neuroinflammation and recovery. More evidence is needed to fully appreciate the role of cytokine dysregulation prior to injury. Studies that directly increase cytokines prior to injury will give the field more insight into potential mechanism that drive this interaction in the aged brain.

### Oxidative Stress

During cellular respiration, electrons can escape the electron transport chain (ETC) and can either directly combine with oxygen molecules or drive chain reactions to produce highly reactive oxygen radicals. Directly, NOX2 can react with leaked electrons with O_2_ to form the superoxide radical (O_2_•-). Indirectly, growing hydrogen peroxide (H_2_O_2_), which itself can serve as an oxidating species, can react with free iron molecules to create hydroxyl radicals (•OH), often the driver of damaging lipid peroxidation (Ayala et al., 2014). Together, superoxide and hydroxyl radicals, and well as hydrogen peroxide and singlet oxygen act collectively as ROS. In homeostasis ROS are generated and subsequently cleared by the body to prevent damage to tissues. Rising levels of ROS in the body is generally indictive of immune and/or metabolic dysfunction triggered by injury or disease. In both CCI and fluid percussion models of TBI, ROS production peaks 30 min following injury, but can last for several hours and is dominated by superoxide production (Kontos and Wei, 1986; Marklund et al., 2004).

In homeostatic conditions ROS are produced as a byproduct of oxygen metabolism. These free radicals can then become involved in redox signaling and can be neutralized by anti-oxidant enzymes (Eastman et al., 2020). Following injury however, metabolic bursts by microglia as they react to injury produce levels of ROS that cannot be attenuated by normal homeostatic mechanisms. When ROS production outpaces compensatory mechanisms, the increase in ROS results in patterns of damage called oxidative stress. Animal models of moderate TBI have confirmed that oxidative stress is prolonged and multifaceted following injury. Specifically, oxidative stress can damage DNA structure and the side of the brain ipsilateral injury is particularly vulnerable to lipid peroxidation, and metabolic disruption caused by ROS (Mendez et al., 2004; Ansari et al., 2008). This rise in ROS and associated oxidative stress contribute to the secondary injury phase of TBI (Clausen et al., 2004). Post-TBI attenuation of oxidative stress by inhibiting ferroptosis reduced lesion volume and improved MWM performance in a CCI model for moderate TBI (Xie et al., 2019).

These free radicals can also be produced by exogenous stressors other than TBI. Environmental factors such as pollution and UV exposure, cigarette smoking and alcohol ingestion, and even prior infections or particular pharmaceuticals such as non-steroidal anti-inflammatory drugs (NSAIDs) can all increase production of ROS (Bhattacharyya et al., 2014). Relevantly, ROS load also increases over the course of a person's lifetime, in part due to exposure to the aforementioned agents. Therefore, both pre- and post-TBI stressors have the potential to generate more free radicals in addition to those produced by the injury, increasing the overall load of oxidative stress on neural tissues and leading to some of the deleterious effects observed following secondary stressors. For this reason, ROS load at time of injury may have a significant role in the severity and longevity of secondary injury. This has been investigated specifically in the context of age at time of injury. In an aging mouse model of CCI, Kumar et al. (2013) found that 24-month aged TBI animals simultaneously had increased NOX expression and decreased gene expression of superoxide dismutase-1 (*Sod1*), an endogenous antioxidant, compared to young injured mice (Kumar et al., 2013). Similar CCI models comparing young and aged rats investigated the functional consequences of these gene changes, finding that 4-HNE, a marker a lipid peroxidation, remained significantly elevated 7 dpi compared to young injured animals (Shao et al., 2006).

As previously mentioned, oxidative stress is thought to contribute to immunosenescence with age. The NOD-, LRR-, and pyrin domain-containing 3 (NLRP3) inflammasome can react to both ROS themselves and DNA damaged by ROS to induce transcription of pro-inflammatory cytokines (Zhou et al., 2011). Therefore, attenuating ROS production following TBI is an attractive therapeutic route. Reducing TBI pathogenesis and improving outlook by intervening in free radical production prior to injury shows promise. NOX enzymes in particular have received attention as they are a potent source of superoxide following injury. Inhibition of NOX2 activity prior to moderate TBI induction *via* weight drop attenuated both BBB disruption and neurological deficits. Additionally, in treated TBI animals there was a decrease in TUNEL-positive cells indicating that apoptosis was also attenuated (Lu et al., 2014). Full NOX2 signaling inhibition may not be necessary to reduce ROS load and oxidative stress in the brain. Cui et al., found that administration of a vitamin-D metabolite, stimulated expression of vitamin-D receptor and down-regulated NOX2. Animals receiving this treatment following CCI improved on neurological exams quicker than untreated, injured subjects, had improved performance on MWM, and improved hippocampal neuron survival (Cui et al., 2017). Critically, these results demonstrate both that the therapeutic window for ROS-attenuating treatments is valid post injury, and that full NOX inhibition is not necessary, thus allowing for the preservation of redox signaling pathways. However, neither study was performed on animals that were currently experiencing exacerbated ROS production or oxidative stress at time of injury and it is unclear how these interventions may interact in an inflammatory environment.

### Mitochondria

#### Aging as a Source of Pre-TBI Mitochondrial Dysfunction

As organisms age, mitochondria become less efficient with impaired oxidative phosphorylation (OXPHOS) leading to depressed ATP production (Ferrándiz et al., 1994). Mitochondria can sense and respond to endogenous cytokines, and can be damaged by ROS which can stem from endogenous and exogenous sources during the aging process. Inflammatory cytokines IL-1β, IL-6 and TNF-α have demonstrated that they can impair mitochondrial energy production in non-CNS tissues (Tatsumi et al., 2000; Kowluru et al., 2011; Kastl et al., 2014; Tyrrell et al., 2020). While the specific mechanism as well as whether the effects are present in microglial mitochondria isn't clear, Kastl et a., found that TNF-α treatment on Hepa1-6 (mouse) and Huh-7 (human) liver cell lines *in vitro*, depolarized mitochondrial membranes, decreased ATP production, and increased ROS release. Similar mitochondrial deficits have been described in BV2 cells treated with LPS, indicating that cytokines produced by TLR activation may directly signal changes in mitochondria function and efficiency (Voloboueva et al., 2013; Molagoda et al., 2021). As previously mentioned, transient cytokine levels increase in the brain with age, thus posing a greater risk to mitochondrial heath.

**Figure 2:**
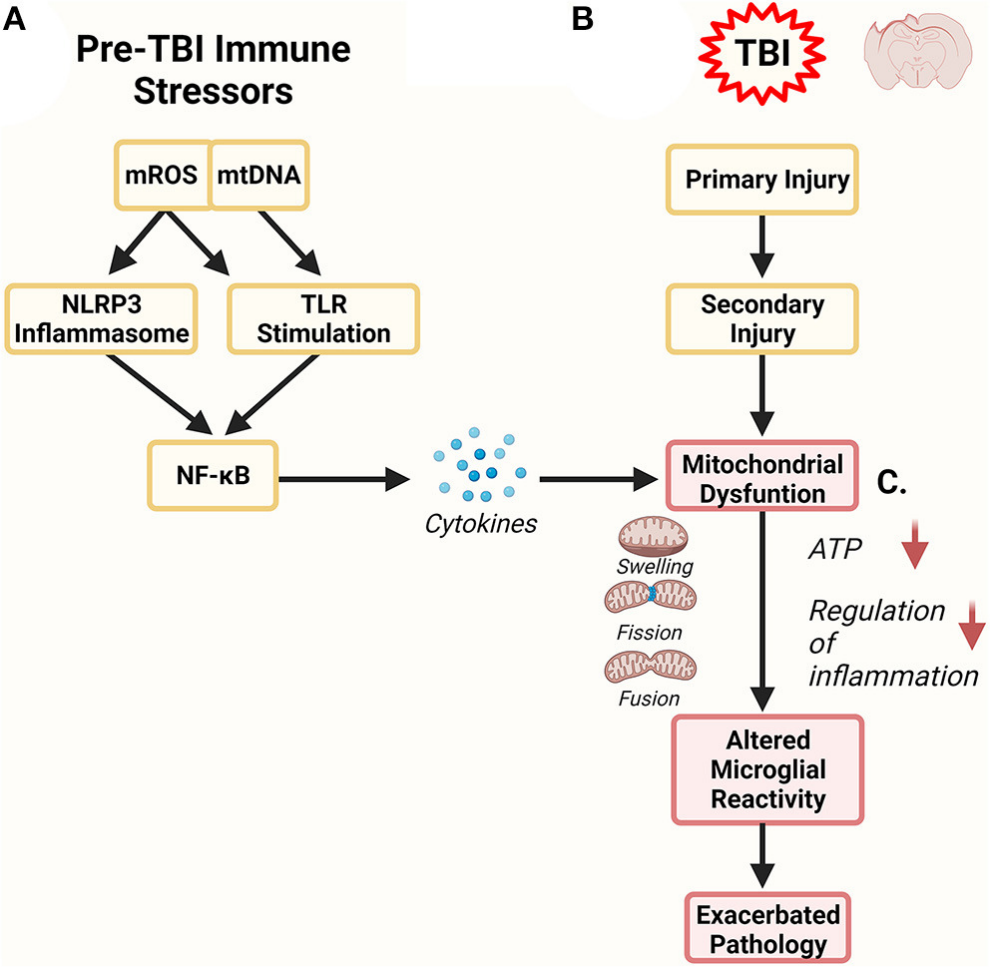
Mitochondrial Role in Inflammation. Mitochondria are both a target of inflammation and potentiate an inflammatory response. **(A)** Prior to TBI, inflammation can alter mitochondrial function, leading to increased release of mitochondrial DNA (mtDNA) and mitochondrial reactive oxygen species (mROS). Both of these serve as damage-associated molecular patterns (DAMPs) and can further stimulate inflammatory pathways *via* the NOD-, LRR- and pyrin domain-containing (NLRP3) inflammasome and toll-like receptors (TLR). In these pathways NF-kB activation stimulates the production of more inflammatory regulators like cytokines which can directly affect mitochondrial function. **(B)** Traumatic brain injury has primary and secondary phases of injury. The secondary phase of injury is associated with maladaptive neuroinflammation. One component of this phase are dysfunctional mitochondria. **(C)** Pre-TBI mitochondrial dysfunction can combine and synergize with mitochondrial dysfunction observed in secondary injury. Altered mitochondrial dynamics can exacerbate mitochondrial health, impairing their ability to properly regulate microglial reactivity. Dysregulation of microglial reactivity to TBI could have profound consequence on the severity and longevity of secondary injury and could represent a link to future neurodegeneration.

Mitochondria represent an important link between ROS production, and the neurodegeneration and metabolic dysfunction observed after TBI. Briefly, the classical function of mitochondria is to produce energy in the form of ATP for cells to use by the process of OXPHOS. In homeostasis, mitochondrial metabolism produces mitochondrial ROS (mROS), however, mROS production at this level can be mitigated by endogenous enzymatic and non-enzymatic antioxidants (Rizzo et al., 2010; Brand et al., 2013). These low levels of ROS are an important part of redox signaling pathways (Schieber and Chandel, 2014). ROS production can be increased by a variety of endogenous and exogenous factors. Endogenous ROS production generally stems from increased metabolic rates in cells, particularly immune cells responding to infection, damage, psychological stress, and aging. Exogenous ROS production can happen by radiation or when environmental pollutants, certain foods and beverages like alcohol, and heavy metals enter the body and are metabolized with ROS being generated during that process (Pizzino et al., 2017). When production of ROS outpaces its removal, the remaining ROS can damage proteins, lipids and DNA which is called oxidative stress. Mitochondria can both be a source of ROS and a target for oxidative stress (Figure 2). ROS in the mitochondria can damage mtDNA by oxidizing nucleic acids leading to lesions in the mtDNA strand (Singh et al., 2019). The rate of mutations for mitochondrial DNA (mtDNA) is estimated between 10 and 1,000x higher than eukaryotic DNA (Beckman and Ames, 1999; Haag-Liautard et al., 2008; Mingroni-Netto, 2021). While mROS generated by the nearby ETC are not the sole culprit of mtDNA damage, they likely contribute. This process feeds back upon itself as mtDNA damage leads to higher mROS generation which in turn damages the mitochondria, decreasing metabolic output (Hahn and Zuryn, 2019).

Aging itself also impacts mitochondrial health. As organisms age, mitochondria accumulate damage due to oxidative stress including lesions to their DNA that result in impaired OXPHOS, reduced ATP generation, higher production of ROS, and impaired dynamics (Bokov et al., 2004; Chistiakov et al., 2014). In aged animals, mitochondria become swollen and branched, which may be due to either increases in fusion or decreases in fission dynamics (Mai et al., 2010; Leduc-Gaudet et al., 2015). These morphological changes may be an attempt to protect respiratory function in mitochondria (Stauch et al., 2014). However, other data from both mouse and rat models show that aspects of OXPHOS metabolic efficiency decreases with age, likely as a result of both morphological changes, accumulating mtDNA mutations, and oxidative stress (Harmon et al., 1987; Ferrándiz et al., 1994). Together research indicates that mitochondrial dysfunction is present during the aging process and mitochondria may be vulnerable to any secondary sources of stress (Sun et al., 2016; Agrawal and Jha, 2020). This is particularly important in the context of pre-TBI aging. Mitochondria that are dysfunctional in microglia due to aging processes may be further exacerbated by inflammation following a TBI, impair microglial reactivity, and thereby contribute to secondary injury. However, the effects of pre-TBI mitochondrial stress on TBI pathogenesis in aging models are not yet classified and should be examined further.

A number of immune stressors including infection, endogenous physiological stressors such as pollution and toxins, Aβ and certain genetic variants that are commonly associated with AD can all individually affect mitochondrial health and metabolic output (Bhattacharyya et al., 2014; Agrawal and Jha, 2020). As an individual ages, the likelihood of exposure to any of these factors that affect mitochondrial health increases. The effect of inflammatory signaling on microglial mitochondria still needs to be examined further. However, there is some evidence that Aβ induces an influx of Ca^2+^ into BV2 and mouse microglial mitochondria *in vitro*, deceasing ATP production, increasing mROS release and triggering mitochondria-driven apoptosis (Xie et al., 2017). These findings indicate that mitochondria are likely sensitive to the internal inflammatory cascade in microglia and further research into this interaction will help to clarify the effects of inflammation on mitochondrial health within the CNS immune system.

### Mitochondrial Control of Inflammation Bioenergetics

Mitochondria and their products regulate microglial reactivity in two distinct ways. First, mROS and mtDNA are both recognized as DAMPS and can trigger the production of pro-inflammatory cytokines *via* NF-κB stimulation. Both can trigger the NLRP3 inflammasome and mtDNA can additionally activate TLR9, inducing pro-inflammatory signaling cascades (Zhou et al., 2011; Goulopoulou et al., 2012; Mathew et al., 2012). Second, mitochondrial function has a multifaceted role in the regulation of microglial bioenergetics. Metabolically, GLUT1 drives a shift from OXPHOS to glycolysis in microglia to support the cell growth and rapid production of cytokines and ROS that are needed to react to an immune challenge (Everts et al., 2014; Wang et al., 2019). While glycolysis takes place in the cytoplasm, there is evidence in microglial BV2 cells that mitochondria help facilitate this metabolic shift *via* mitochondrial glucose-regulated protein 75/mortalin (GRP75) (Voloboueva et al., 2013). Additionally, mitochondrial density increases in microglia activated by IFN-γ or LPS, despite playing less of a role in metabolism, indicating that they play additional roles in stimulating microglial reactivity (Banati et al., 2004). One proposed role is that mitochondrial proteins play a direct role in activating transcription of pro-inflammatory products. Castanier et al. (2010), found that regulators of mitochondrial fusion proteins mitofusin 1 (MFN1) and optic atrophy 1 (OPA1) increased activation of both NF-κB and IFN-β promoter in HeLa cells. Overall, mitochondria have an important role in the regulation of inflammation both in controlling metabolism and in inducing inflammatory cascades. Therefore, if mitochondrial function is disrupted by a pre-TBI immune stressor, this could impair microglias' ability to properly respond to an injury.

### Role of Mitochondria in Secondary Injury

Mitochondrial disruption after injury may be a contributing factor to secondary injury. Therefore, a TBI which increases ROS in the brain may also lead to cascading damage to mitochondria, impacting metabolism and cellular health during the secondary phase of injury. Additionally, following TBI, mitochondria display altered functional dynamics which can lead to increased ROS load and decreased metabolic output (Hiebert et al., 2015). Moderate lateral FPI in rats caused structural and functional mitochondrial damage after injury with the number of mitochondria present in the ipsilateral cortex significantly decreased correlating with a decrease in both hippocampal and cortical ATP production 24 h-post injury (Lifshitz et al., 2003). Moderate weight drop TBI also showed a decrease in OXHPOS proteins in whole brain mitochondria, indicating that the neuroprotective shift from OXPHOS to glycolysis may be impaired following injury (Chen et al., 2016). Mitochondrial dynamic can roughly be broken down into two processes, fusion and fission. In homeostasis, mitochondria undergo constant processes whereby they divide and fuse to other mitochondria (Xie et al., 2018). This is thought to be protective, allowing dysfunctional mitochondria to fuse to healthy mitochondria to have function restored where fission can cleave chronically dysfunctional mitochondria for them to be recycled through mitophagy (Youle and van der Bliek, 2012; di Pietro et al., 2017). These processes are driven by a family of proteins namely, dynamin-related protein 1 (DRP1), mitochondrial fission 1 protein (FIS1), MFN1/2, and OPA1 (Cipolat et al., 2004; Naotada et al., 2004; Fonseca et al., 2019). Under acute stress, mitochondrial fusion is upregulated as a protective measure, however, chronic stressors and TBI tip the scale, inducing high levels of mitochondrial fission which can lead to apoptosis (Youle and Karbowski, 2005; Youle and van der Bliek, 2012).

These discoveries have led researchers to hypothesize that dysregulation of mitochondrial dynamics contributes to secondary injury (Dobrachinski et al., 2017). This dysregulation following injury have been well-documented in several models of TBI although differences in the exact nature of dysregulation are found between injury severities (Fischer et al., 2016; di Pietro et al., 2017). In a rat CCI model for moderate TBI, Salman et al. (2020) found that the mitochondrial fraction of DRP1, the major protein necessary for fission increased while decreasing in the cytosolic fraction, indicating recruitment of DRP1 to the mitochondria post injury. Consequently, several studies have sought to intervene on DRP1 activity post-TBI in order to alleviate components of secondary injury. So far, therapeutics have focused on preventing pre-TBI mitochondrial dysregulation and have found success. Salman et al., also found that treatments with melatonin post-TBI had several positive benefits. Functionally, they observed a decrease in fission activity confirmed by electron microscopy; this correlated with an increase in the number of viable neurons in the treated group. As a result, the treated rats also had improved performances on a variety of motor-behavior tasks (Salman et al., 2020). Previous papers have reported similar findings using various pharmacological interventions to prevent DRP1 driven fission post-TBI (Wu et al., 2016, 2018; Song et al., 2019).

## AD Genetic Risk Variants Associated With Microglia

AD is a neurodegenerative disease that is traditionally marked by the accumulation of two protein aggregates, amyloid beta (Aβ) plaques, and neurofibrillary tangles (NFT). In AD, amyloid precursor protein (APP) is cleaved by β and γ-secretases and produces 40 and 42 amino acid-length soluble oligomers. In disease conditions these oligomers can aggregate and form plaques (Aβ42), or localize into the vasculature and cause cerebral amyloid angiopathy (CAA) (Aβ40) (Serrano-Pozo et al., 2011). NFTs form when tau, a microtubule stabilizing protein, phosphorylates (ptau) causing it to dissociate from the microtubule and misfold. These misfolded tau proteins aggregate into NFTs where they accumulate inside neurons until cell death occurs. When affected neurons die, NFTs can travel and enter nearby neurons, contributing to the topographical spread of AD (Serrano-Pozo et al., 2011).

Both amyloid plaques and ptau have a consorted effect on microglial function and neuroinflammation. Aβ can bind to a variety of receptors on microglia to induce either uptake of Aβ into the microglia, or induce an inflammatory microglial response. Induction of a pro-inflammatory microglial response can be achieved by the binding of Aβ to the scavenger receptors MARCO and CD36, RAGE, TLR2 and TLR4, formyl peptide receptor 2, and chemokine-like receptor 1 (CMKLR1) (McDonald et al., 1998; Yu and Ye, 2014). As part of the inflammatory response to Aβ, Baik et al. (2019) also found that Aβ can also trigger a metabolic shift in microglia from OXPHOS to glycolysis. As previously mentioned, this shift is observed in response to other acute inflammatory events and is thought to be protective as a way to supply more ATP to microglia undergoing a shift from a surveying to reactive phenotype (Lauro and Limatola, 2020; Takeda et al., 2021). These findings indicate that Aβ fibrils can induce a strong inflammatory response in microglia.

Tau also interacts with microglia to stimulate inflammation. Microglia appear to react to ptau in both humans and in mouse models for tauopathy (Bellucci et al., 2004; Sasaki et al., 2008). In a *P301s* (MAPT) mouse model for tauopathy, microglial activation and astrocytic gliosis preceded NFT formation by at least 3 months lending further evidence that microglia are activated in early tau pathogenesis (Yoshiyama et al., 2007). Additionally, recent studies have discovered that non-phosphorylated tau can also stimulate microglial activation *via* the p38 pathway reducing total tau either *via Mapt* KO or *via* antibody treatment demonstrate promise in attenuating microglial response and reducing inflammation (Maphis et al., 2015; Perea et al., 2018; Zilkova et al., 2020). Pathological tau tangles can trigger further immune responses as well. Earlier research found associations between microglial density and NFT presence and more recent studies have indicated that NFTs may induce microglial activity by triggering compliment pathways (DiPatre and Gelman, 1997; Shen et al., 2001). Specifically, inhibition of C3a receptor rescued both tau pathology and hippocampal-dependent memory in *P301s* mice (Litvinchuk et al., 2018).

The inflammatory cascade of TBI interacts with AD pathology. Some earlier research by Nakagawa et al., found that 4 month-old PDAPP mice given TBI *via* CCI (5 m/s, 1 mm depth) developed less Aβ plaques in the hippocampus and cingulate cortex by 9 and 12 months compared to sham injured mice (Nakagawa et al., 1999). Later, Nakagawa et al., also found that TBI may alleviate antecedent Aβ pathology. Two year-old PDAPP mice, with existing Aβ plaques, given CCI (5 m/s, 1 mm depth) actually exhibited a decline in Aβ plaques following injury (Nakagawa et al., 2000). More recently, Garcia et al., found that low-level repetitive blast injury in APP/PS1 mice decreased Aβ plaque and resulted in improved performances on elevated plus maze, light/dark emergence, and open field test, indicating decreased anxiety-like behavior. Mice that received the repetitive blast injury also had improved performance in NOR and Barnes maze tests, indicating improved cognition (Perez Garcia et al., 2021). Of these studies the 2000 study by Nakagawa et al., is of particular interest. Their animal model, PDAPP mice, begin displaying AD pathology at 6 months, meaning these animals certainly had ongoing AD-related pathology at the time of injury. Similar findings have not recently been published but the findings do warrant further examination into how AD-related pathology and neuroinflammation in this model may precondition the brain prior to TBI. Most recent data indicates that in these models moderate TBI pathology is exacerbated, and AD pathogenesis is accelerated post-injury (Washington et al., 2014; Gerson et al., 2016; Kokiko-Cochran et al., 2018; Zyśk et al., 2019). However, these studies focus on the effects of pathogenic hallmarks of AD on TBI pathology, and less so on how AD-related changes in the CNS immune system prior to injury may affect outcome. Genome-wide association studies (GWAS) have identified variations in several inflammation-related genes that associated with some amount of risk or AD development. Of these genes, variations in *APOE* and *TREM2* have high levels of risk for disease but are less common. More common variations in genes *CLU, CD33, ABCA*7, CR1, HLA-DRB1, INPP5D, and MS4A convey a smaller risk for AD development but are believed to play roles in the regulation of inflammation. These genes affect a variety of microglial functions including cell growth, trafficking, phagocytosis, and chemokine response. Possession of one or several of these genetic risk factors can impair these microglial functions, possibly damaging the brain's ability to respond to injury. Therefore, we will now review the role these genes play in the regulation of microglia and how polymorphisms affect said function. Additionally, where applicable we will highlight literature investigating how these genetic risk factors influence TBI pathology.

### APOE4

Apolipoprotein E (APOE) plays a role in cellular metabolism and genetic variations that affect protein function have a downstream effect on mitochondrial dynamics and metabolic function. APOE is a lipoprotein that plays a supporting role in cellular metabolism by transporting lipids, notably cholesterol, from astrocytes to neurons. APOE likely plays a role in regulating neuroinflammation that will be explored further, but also has genetic variants like the *APOE-*ε*4* (APOE4) isoform that are tied to an increased risk for developing late onset AD (LOAD). Current evidence suggests that APOE may play a role in the regulation of glial homeostasis, and alleles like the ε*4* may disrupt this homeostasis and lead to an altered inflammatory response to challenges. Transcriptomic analysis of *Apoe3* and *Apoe4* mice after a moderate CCI injury found that genes related to innate immunity were upregulated in the ε*4* mice compared to the ε*3* mice, indicating that APOE4 may convey a heightened pro-inflammatory response (Castranio et al., 2017). *Ex-vivo* stimulation of human blood samples found that production of multiple pro-inflammatory cytokines including TNF-α, IL-1β, IL-6, IL-17, and IFN-γ were significantly increased in carriers of at least one ε*4* allele compared to ε*3/3* carriers (Gale et al., 2014). Similar findings are observed in mouse models where LPS stimulation increases TNF-α and IL-6 levels in serum and brain tissues and IL-1β in the brains of *Apoe4* mice (Lynch et al., 2003; Zhu et al., 2012).

Recent studies tend to agree that APOE play an important role in both microglial motility and phagocytosis, however there is conflicting evidence on how the ε*4* allele impacts these functions. In 2011 Cudaback et al. (2011) used *Apoe*^−/−^ mice with targeted humanized *APOE2, APOE3*, and *APOE4* and found that APOE4 decreased C5a and ATP stimulated migration by about 49 and 61%, respectively, of the maximum response measured in APOE3 mouse microglia. More recently, another study confirmed these findings in a preclinical mouse model where ε3 phospholipids induced greater microglial activation and faster migration to injected Aβ compared to ε4 phospholipids (Fitz et al., 2021). In contrast, Muth et al., found that microglial from the *Apoe4* transfected mice actually increased microglial migration in response to a C5a chemoattractant. This group additionally looked at phagocytosis activity and found that while APOE4-expressing microglia had increased phagocytosis of apoptotic neurons while uptake of accumulating Aβ decreased (Muth et al., 2019). Overall, APOE and its lipoprotein products do appear to play a role in microglial activation and response to immune challenge. If an individual possesses one or two ε4 alleles this could alter the ability of microglia to properly respond to injury. With this in mind, clinical evidence indicates that *APOE4* possession is predictive of worse outcomes after TBI (Friedman et al., 1999; McFadyen et al., 2021). Critically, Mannix et al., found the APOE4 may affect TBI outcome in an age-dependent manner. In a mouse CCI model for severe injury MWM performance was the same 2 weeks after injury between wild-type and *Apoe4* mice. When mice were tested again 6 months after injury *Apoe4* mice had significantly worse performance indicated by increased latency to platform times (Mannix et al., 2011). These data indicate that APOE4 influences TBI outcome and may interact with age after injury to do so. A variety of APOE4 dependent cellular effects have also been observed in various TBI models including, increases in pro-inflammatory cytokines and ptau, enhanced microglial activation, and blood brain barrier repair impairment (Main et al., 2018; Giarratana et al., 2020).

### TREM2

The role of TREM2 in the regulation of inflammation and the effect of variants on TBI pathology have been well-documented. TREM2 is a receptor expressed on the cell surface of microglia involved in anti-inflammatory signaling in the CNS. TREM2 has several ligands including apolipoproteins like APOE, and Aβ that, when bound, induce a variety of effects *via* its adapter protein DAP12. TREM2 activation has been shown to both increase microglial phagocytic activity and promote survival and proliferation of microglia (Yuan et al., 2016; Zheng et al., 2016). TREM2 activity plays an important role in AD pathogenesis. The *R47H* variant is a loss-of-function mutation of TREM2 and presence of this variant is associated with a two to four-fold increase in risk to develop LOAD (Gratuze et al., 2018). Currently, much evidence points to loss of function mutation or haploinsufficiency of TREM2, directly alters microglial activity thus impairing the brain's ability to combat AD pathology *via* neuroinflammation. As previously stated, TREM2 plays an anti-inflammatory role decreasing production of pro-inflammatory cytokines and promoting anti-inflammatory cytokine release, while helping maintain cellular metabolism in microglia (Takahashi et al., 2005; Ulland et al., 2017). Despite its prominent role in regulating inflammation there is a lack of research on the role of TREM2 in TBI. TREM2 deficiency has been examined in a mouse model for moderate lateral FPI finding that in the acute phase after injury macrophage activity and *Tnf-*α transcription were attenuated by TREM2 deficiency. In the chronic phase, memory deficits measured by Y maze and MWM were reversed by TREM2 deficiency, correlating with a decrease in hippocampal atrophy (Saber et al., 2017). To this date it is unclear whether the *R47H TREM2* variant will provide neuroprotection like TREM2 deficiency. It is also possible that the impaired metabolism and anti-inflammatory signaling found in *TREM2-R47H* impair the inflammatory profile of the CNS prior to injury. This maybe especially true if animals possessing the variant are exposed to a preinjury immune challenge.

### CLU

The *CLU* gene codes for clusterin, also known as apolipoprotein J, a glycoprotein that is involved in a variety of lipid transport and chaperone functions in the CNS. Clusterin interacts with the immune system in disease states by serving as a sensor of oxidative stress through interactions with TGF-β, preventing Aβ aggregation by sequestering oligomeric Aβ, and serving as a TREM2 ligand (Narayan et al., 2011; Athanas et al., 2015; Yeh et al., 2016). *Clu*-KO model have showered exacerbated pathology in both MCAO and AD models and *CLU* is upregulated following TBI, indicating that normal clusterin function is neuroprotective (Wehrli et al., 2001; DeMattos et al., 2004; Imhof et al., 2006; Troakes et al., 2017). Currently, 6 different single nucleotide polymorphisms (SNP) have been linked to AD risk. The most thoroughly investigated SNP is rs11136000 which as two alleles, C and T. Of the two, T is thought to be neuroprotective while the C allele conveys a 1.6 fold increased risk for AD (Foster et al., 2019). Differences in clusterin's efficiency in serving as a sensor of oxidative stress, its Aβ sequestration, or its TREM2 binding affinity could explain its contribution to AD pathology. These same changes could potentially injure the brain's ability to respond to injury as well and should be investigated further.

### CD33

CD33 is an inhibitory transmembrane receptor expressed on myeloid cells that can be stimulated by a variety of glycoproteins and lipids. When stimulated, CD33 has an inhibitory role on microglial functions in the brains including phagocytosis and cytokine release (Zhao, 2019). GWAS have identified two CD33 SNPs that increase risk of AD, *rs3865444* and *rs12459419* (Lambert et al., 2013; Malik et al., 2013). Specifically, the allele *rs3865444C* increases CD33 expression in AD. This increased CD33 expression in turn, further inhibits microglial phagocytosis and clearance of Aβ (Griciuc et al., 2013). While studies have not looked at how this risk variant may impair microglial function in a TBI context, other studies have found decreased microglial phagocytosis in aged mice that received moderate CCI, correlating with exacerbated neurological deficits in the acute phase (Ritzel et al., 2019). This microglial dysfunction is likely influenced by the heterogenous factors that lead to immunosenescence; however, it can also indicate that increased CD33 expression by the *rs3865444C* allele could complicate TBI pathology and recovery by downregulating microglial phagocytic activity.

### ABCA7

ATP-binding cassette transporter A7 (ABCA7) is a membrane transporter protein expressed on microglia. This protein is highly involved in transporting energy and nutrients across the cell membrane and thus plays a critical role in maintaining microglial homeostasis. Additionally, ABCA7 contributes to regulating microglial phagocytosis and clearing of apoptotic cells (Jehle et al., 2006). Multiple SNPs have been located that confer AD risk, all mutations are associated with ABCA7 loss of function (Reitz et al., 2013; Allen et al., 2017). Loss of ABCA7 function has been directly implicated in microglial dysregulation. ABCA7 haplodeficiency, impaired the ability of microglia to respond to LPS stimulation. Abca7^+/−^ mice had lower cytokine responses and, when crossed with an APP mouse line, demonstrated microglial Aβ accumulation (Aikawa et al., 2019). Loss of ABCA7 function could also impact phagocytosis regulation. Dysfunctional cytokines signaling and phagocytosis responses in microglia seem to present a mechanism for how these variants confer AD risk. Since these microglial functions are necessary to respond to TBI, investigating how these variants impact TBI pathology will be important.

### C1

Complement receptor 1 (CR1) binds complement factors C3b and C4b. Reactive microglia upregulate CR1 expression and activation of the receptor can induce apoptosis (Crehan et al., 2013). Products of the complement system can regulate various microglial activities such as chemotaxis and debris clearance. Complement can also play both a protective role against neurotoxicity and neuron death and a harmful role in inflammation by promoting inflammation and Aβ aggregation (Bonifati and Kishore, 2007). Nine CR1 SNPs, have been identified to confer AD risk with most resulting in differential expression of CR1 (Lambert et al., 2009; Zhu et al., 2014). In AD, the CR1 variants appear to affect the ability of microglia to clear Aβ (Brouwers et al., 2011). However, the exact nature of microglial dysregulation is not well-documented for these SNPs. As CR1 may play dueling roles in regulating inflammation, it is difficult to predict how these polymorphisms could alter the glial response to injury.

### HLA-DRB1

HLA class II histocompatibility antigen, DRβ1 (HLA-DRB1) is member of MHCII. It is expressed on antigen presenting cells and plays a role in both innate and adaptive immunity. Currently GWAS have identified at least two SNPs associated with increased risk for AD, rs9271058 and rs9271192 (Lu et al., 2017; Yan et al., 2020). Recent information has found that HLA-DRB1 is upregulated by microglia in AD, however the exact mechanism by which HLA-DRB1 interacts with microglial reactivity remains elusive (Villegas-Llerena et al., 2016). Therefore, it is currently unknown what effects these genetic variants confer onto microglia. However, recently a role for HLA-DRB1 in tau processing in TBI has been suggested. Demock and Kornguth suggest that intracellular tau accumulation is driven by the binding of extracellular tau to membrane-bound HLA, which then undergoes endocytosis (Demock and Kornguth, 2019). Further research should be conducted to investigate this hypothesis and determine whether variations in HLA-DRB1 differentially affect tau processing in both AD and TBI contexts.

### INPP5D

INPP5D codes inositol polyphosphate-5-phosphatase, also known as SHIP1, a phosphatase enzyme. SHIP1 plays a regulatory role in immune activation, serving as a “brake” against inflammatory signals. In part, this involves the phosphorylation of PIP_3_ by SHIP1 which downregulates downstream inflammatory pathways including MAPK and NF-κB. For this reason, SHIP1 is considered an important anti-inflammatory and anti-phagocytic regulator (Pauls and Marshall, 2017). The SNP *rs35349669* has been identified is associated with AD risk (Lambert et al., 2013). WT SHIP overexpression have shown altered outcomes to LPS stimulation, inhibiting TNF-α production and reducing TLR4/MyD88 interaction (An et al., 2005). Additionally, SHIP1 inhibits downstream TREM2 signaling (Peng et al., 2010). These findings are relevant as the AD associated SNP is hypothesized to increase overall expression of SHIP1. As previously described, TNF-α, TLRs, TREM2 and the ERK/MyD88 pathway are all implicated in the immune response to TBI. Overexpression of SHIP1 by this variant inappropriately suppressing immune pathways is a likely candidate for the mechanism of increased AD risk, interfering with these pathways could alter the inflammatory response to TBI as well and should be investigated.

### MS4A

Membrane-spanning 4-domain subfamily A (MS4A) is a transmembrane receptor expressed on microglia that has been implicated in signaling and endocytosis although its exact role in microglia remains undefined (Cruse et al., 2015; Efthymiou and Goate, 2017). Several SNPs have been identified that confer an increased risk for LOAD including rs610932, rs4938933, and rs670139 (Naj et al., 2011; Zhu et al., 2016). Of these rs670139 and are associated with the member gene MS4A4A which has a role in TREM2 processing. Previous research found that a different SNP, rs1582763, on MS4A4A altered soluble TREM2 (sTREM2) levels in the CSF and GWAS associated this variant with decreased risk for AD (Deming et al., 2019). As rs670139 is associated both with MS4A4A and increased AD risk, it is possible that this variant has a different effect on sTREM2 production. The role of sTREM2 in TBI pathology is not understood however, analysis of cerebral spinal fluid (CSF) in former football players did find correlations between higher sTREM2 ad higher total tau (Alosco et al., 2018).

### Microglial Mitochondrial Are Critical for AD Pathogenesis

Mitochondrial dysfunction appears early in the pathogenesis of AD in several forms. Structurally, mitochondria in early AD brains display altered fission/fusion dynamics, similar to those observed following TBI. A study of human AD patients with varying stages of AD, found several key changes in the expression of fission and fusion related proteins. Regardless of pathogenesis stage, real time qRT-PCR found that expression of fission genes increased while expression of fusion genes decreased, these findings were corroborated by immunoprecipitations showing increases in the fission proteins DRP1 and FIS1, and decreases in fusion proteins MFN1/2, and OPA1 (Manczak et al., 2011). TEM studies in APP mice found that mitochondria fragment in both the soma and in neurites and pure number of mitochondria significantly decreases in neurites (Calkins et al., 2011). A similar study using several FAD mouse models for AD confirmed increased fission dynamics, including a novel phenotype displaying fission arrest, where mitochondria would begin but not finish the fission process creating a string of fragmented mitochondria (Zhang et al., 2016). These findings have coincided with discoveries that Aβ interacts with DRP1, and that introduction of oligomeric Aβ ligands to hippocampal cells *in vitro* can drive redistributions in mitochondria and increases in *Drp1* gene expression (Wang et al., 2009; Calkins et al., 2011; Manczak et al., 2011).

So far, these findings have been localized within neuronal mitochondria. Mitochondria in microglia may be playing an enhanced role in this pathogenesis. If mitochondrial health is exacerbated in microglia in a similar fashion, this could have the added detriment of impairing the inflammatory response to AD. TREM2 and APOE can each influence mitochondrial activity, therefore variations in these proteins may have downstream effects on both mitochondria and the mitochondrial regulation of inflammation. Specific links between APOE4 and changes in mitochondrial dynamics have been found in both clinical and pre-clinical settings. Early clinical evidence found that levels of α-ketoglutarate complex (KGDHC), a mitochondrial metabolic enzyme, were diminished in AD patients with the *APOE4* variant (Gibson et al., 2001). These results indicated APOE4-dependent deficits in mitochondrial function that correlated with higher levels of oxidative stress in the brains of *APOE4* patients (Ramassamy et al., 1999). Recent clinical evaluation of brain tissue from *APOE4* carriers and non-carriers found differential expression of mitochondrial dynamics-related proteins. The study found downregulation of proteins involved in mitochondrial biogenesis and dynamics in the *APOE4* carriers group including; antioxidant proteins SIRT3 and SOD2, as well as dynamics proteins DRP1 and MFN1/2 (Yin et al., 2020). However, there are several drawbacks to this study that animal studies will be able to control for. As previously discussed, expression levels of these same proteins are also affected by age, disease exposure, and other AD risk variants. While groups in the Yin et al., study had similar mean ages (carriers, 84.6; non-carriers, 88.5), and similar prevalence of AD diagnoses, interactions between age and disease are difficult to appropriately control for. Additionally, patients were not screened for additional variants such as the *TREM2-R47H* risk variant. As APOE can serve as a ligand for TREM2, which has its own effects of mitochondrial health and function, it is difficult to assess how these factors could interact and impact results.

Limited researched has been conducted to investigate exactly how *APOE* alleles including *APOE4* interact with mitochondria to influence their dynamics. However, mouse models for *APOE4* have indicated a direct relationship between possession of the risk factor and mitochondrial dysfunction. A study of 4-month old *Apoe4* transgenic mice found increases in MFN1 and decreases in DRP1 protein levels. Through transmission electron microscopy analysis, the investigators found that mitochondria in the *APOE4* animals were elongated with less dense cristae, confirming the functional effect that differential MFN1 and DRP1 levels had on mitochondrial dynamics (Simonovitch et al., 2019). While an exact mechanism is still unknown, previous analysis of human brain tissue found that *APOE3/4* individuals had increased localization of APP in mitochondria where accumulation could impair mitochondrial function (Devi et al., 2006). While other studies have investigated how various *APOE* alleles may affect the pathogenesis of TBI, more research is needed that specifically investigates how mitochondrial metabolic function may be impaired in *APOE4* models and what implications this could have for TBI pathology. Future studies in more complex models of AD such as the hApp/Apoe4/Trem2^*^R47H model could provide an interesting look into how interaction between these genetic risk factors could impair mitochondria prior to injury.

Recent investigations have found correlations between the *TREM2-R47H* risk variant for AD and mitochondrial dysfunction. As previously mentioned, the metabolic capacity of microglia is key in their ability to respond to Aβ in the CNS and TREM2 plays a central role in regulating microglial reactivity. To this end, a study in *Trem*2^−/−^ 5xFAD mice found that AD mice missing TREM2 expression had lower mitochondrial mass in the microglia compared to mice with working TREM2 (Ulland et al., 2017). The authors found that mTOR signaling was altered in the *Trem*2^−/−^ 5xFAD mice, reducing anabolic and metabolic output and decreasing the microglias' ability to respond to Aβ. OXPHOS may also be excessively suppressed by TREM2 deficiency. Pan et al. (2019) found that treating TREM2 deficient mice with sodium rutin boosted OXPHOS, resulting in increased ATP production and a greater ability of microglia to clear Aβ. These data reveal a role for TREM2 in regulating microglial activity through mitochondrial efficiency. If microglia in an organism with loss of TREM2 function have a decreased ability to respond to an immune challenge possessing this variant could have implications the mitochondrial health following TBI as well.

## Conclusion and Future Directions For Research on Pre-TBI Inflammation

TBI related inflammation is a complex, bi-phasic phenomenon. In the early stages, neuroinflammation and microglial activity are beneficial and necessary for clearing debris and supporting the CNS during a vulnerable time acutely post-insult. In contrast, a secondary wave of inflammation triggered by the injury can lead to cellular death and long-term neurological complications. This review has highlighted how these phases of injury are dictated by the state of the CNS immune system at time of injury. The inflammatory profile of the brain is malleable, changing after exposure to previous challenges whether those stem from exogenous or endogenous sources. Studies that have directly looked at pre-injury inflammation have done so through the lens of pre-injury infection. Whether infection occurs *via* in a bacterial (LPS) or viral (Poly I:C) model, the pre-injury stimulation seems to confer neuroprotective preconditioning in the CNS. These findings are interesting but leave several questions. Like TBI, infections have phases to their corresponding immune response each utilizing different aspect of our immune system, innate and adaptive. In studies investigating neuroprotective preconditioning, TBI comes within a week of infectious exposure indicating that this protection may be offered while the CNS's innate immune response is active and responding to infection. Future directions should investigate whether pre-injury infection still provides neuroprotective preconditioning at chronic time points. Doing so will help to elucidate the exact factors that provide the protection and help inform future studies into pre-injury inflammation.

Findings on neuroprotective preconditioning stand in contrast with other evidence that show TBI pathology to be exacerbated by aging. This raises the question, what changes to the inflammatory profile in aging make TBI worse? And how does this differ from the neuroprotective inflammation observed in pre-injury infection models? We went on to take a look at several prominent factors involved in inflammation including cytokine regulation, oxidative stress and ROS production, and mitochondrial health. All these factors display age related deficits and interact with TBI pathology in a unique fashion. Due to this heterogeneity, their effects on microglial reactivity to TBI differ. For instance, cytokine dysregulation with age could drive microglia into a primed state prior to injury. Conversely, mitochondrial dysfunction at the time of injury may impair mitochondrial regulation of inflammatory signaling in microglia. Understanding how dysregulation of each variable impact the immune profile of the brain in the lead up to TBI is important to decern impacts on pathology. Additionally, infection, stress, environmental factors, and genetics can all influence the health of these systems and may compound over a person's life and interact with age. So though post-TBI aging is an important modifier of secondary injury severity and outcome, pre-TBI inflammation may be just as influential despite receiving comparatively less attention. The field would be strengthened by future studies that isolate and modify these variable pre-injury. Doing so would provide the field with clarity on how pre-TBI inflammation and aging impact outcomes.

This review placed emphasis on the role of mitochondria in inflammation, for several reasons. First mitochondria are important actors in the regulation of the inflammatory processes. In homeostasis they are the main source of ATP for surveying microglia and contribute levels of ROS necessary for redox signaling. Under an immune challenge, they control the bioenergetics of microglia, assisting in the transition between reactive phenotypes. Long term deficits in mitochondria's ability to properly regulate inflammation and contribute to metabolism could be a key player in chronic TBI outlook. Second, mitochondria can become a target of inflammation. Their health is impaired in chronically activated microglia and in neurons after severe immune challenges like TBI. Inflammatory factors like cytokines and ROS directly impact mitochondrial function, which in turn can further exacerbate inflammation. Therefore, understanding how they become dysfunctional and how they react to immune challenges once already dysfunctional is important when discussing pre-TBI inflammation. Lastly, mitochondrial dysfunction is present both after TBI and early in the pathogenesis of AD. Currently, clinical evidence suggests there is at least some linkage between TBI and future development of AD (Dams-O'Connor et al., 2016). However, there have also been studies that disagree with this conclusion (Mehta et al., 1999). This maybe be due to both the heterogenous nature of TBI and the plethora of factors that influence inflammation, one of which could well prove to be mitochondria. Studying how cell-specific mitochondria are chronically impacted by TBI is important. Many of the studies described in this review focus on whole brain or neuronal mitochondria. While mitochondrial dysfunction will impact each cell type differently, their responses to inflammation is likely conserved. However, it is still critical for studies to investigate the effect of inflammation on microglial mitochondria to better understand how dysfunction occurs. This information would help to inform how mitochondrial health may then be further exacerbated by TBI inflammation. Better understanding the relationship between inflammation, mitochondria, and microglial reactivity could be key in studying both the exacerbation of post-injury outcomes and also the potential predisposition to future AD development.

When discussing the link between TBI and future AD development, genetics cannot be ignored. Multiple genetic variants have been reviewed that confer some amount for risk for AD. Risk variants like the ε*4* allele for *APOE* and *TREM2-R47H* carry a high risk of LOAD for about 25% of the population. We also reviewed several other inflammation-related genes with variants associated with AD that confer a lower risk but are more common in the population. Many of these associations have only recently been established and therefore, mechanisms of how changes in gene-product function interact with the CNS immune system and AD pathology are generally not well-understood. It stands to reason that if these variations influence the balance of inflammation enough to contribute to AD pathology that their presence may and likely does impact TBI pathology as well. Additionally, while APOE4 and TREM2-R47H each impact mitochondrial function, such effects have not been explored in the other risk variants yet and present an opportunity for further study. Future directions should look for associations between these same genetic risk factors for AD and TBI outcome and prognosis. In addition, basic research can be conducted to investigate how possession of these variants affects inflammation in both health in disease.

## Author Contributions

SH wrote the paper. OK-C provided expertise, wrote, and edited the paper. Both authors contributed to the article and approved the submitted version.

## Funding

This work was supported by a NINDS R01NS109585 to OK-C.

## Conflict of Interest

The authors declare that the research was conducted in the absence of any commercial or financial relationships that could be construed as a potential conflict of interest.

## Publisher's Note

All claims expressed in this article are solely those of the authors and do not necessarily represent those of their affiliated organizations, or those of the publisher, the editors and the reviewers. Any product that may be evaluated in this article, or claim that may be made by its manufacturer, is not guaranteed or endorsed by the publisher.
